# Mixed-matrix membranes with enhanced antifouling activity: probing the surface-tailoring potential of Tiron and chromotropic acid for nano-TiO_2_

**DOI:** 10.1098/rsos.170368

**Published:** 2017-09-06

**Authors:** Avishek Pal, T. K. Dey, A. K. Debnath, Bharat Bhushan, A. K. Sahu, R. C. Bindal, Soumitra Kar

**Affiliations:** 1 Membrane Development Section, Chemical Engineering Group, Bhabha Atomic Research Centre, Trombay, Mumbai 400085, India; 2 Glass and Advanced Materials Division, Bhabha Atomic Research Centre, Trombay, Mumbai 400085, India; 3 Technical Physics Division, Bhabha Atomic Research Centre, Trombay, Mumbai 400085, India; 4 Homi Bhabha National Institute, Anushakti Nagar, Trombay, Mumbai 400094, India

**Keywords:** mixed-matrix membrane, organofunctionalized nano-TiO_2_, Tiron, chromotropic acid, antifouling activity, ultrafiltration

## Abstract

Mixed-matrix membranes (MMMs) were developed by impregnating organofunctionalized nanoadditives within fouling-susceptible polysulfone matrix following the non-solvent induced phase separation (NIPS) method. The facile functionalization of nanoparticles of anatase TiO_2_ (nano-TiO_2_) by using two different organoligands, *viz*. Tiron and chromotropic acid, was carried out to obtain organofunctionalized nanoadditives, F_T_-nano-TiO_2_ and F_C_-nano-TiO_2_, respectively. The structural features of nanoadditives were evaluated by X-ray diffraction, X-ray photoelectron spectroscopy, Raman and Fourier transform infrared spectroscopy, which established that Tiron leads to the blending of chelating and bridging bidentate geometries for F_T_-nano-TiO_2_, whereas chromotropic acid produces bridging bidentate as well as monodentate geometries for F_C_-nano-TiO_2_. The surface chemistry of the studied membranes, polysulfone (Psf): F_T_-nano-TiO_2_ UF and Psf: F_C_-nano-TiO_2_ UF, was profoundly influenced by the benign distributions of the nanoadditives enriched with distinctly charged sites ($-{\text{SO}}_{3}^{-}{\text{H}}^{+}$), as evidenced by superior morphology, improved topography, enhanced surface hydrophilicity and altered electrokinetic features. The membranes exhibited enhanced solvent throughputs, *viz*. 3500–4000 and 3400–4300 LMD at 1 bar of transmembrane pressure, without significant compromise in their rejection attributes. The flux recovery ratios and fouling resistive behaviours of MMMs towards bovine serum albumin indicated that the nanoadditives could impart stable and appreciable antifouling activity, potentially aiding in a sustainable ultrafiltration performance.

## 1. Introduction

The daunting task and challenge of providing adequate and safe drinking water becomes complicated by progressive contamination of accessible freshwater resources with newer contaminants owing to population growth, industrialization and, more importantly, climate change and energy demand. While water is used in all phases of energy production and electricity generation, energy is required to extract, convey and deliver water of appropriate quality for diverse human uses, and then again to treat waste waters prior to their return to the environment. Thus, there exists a strong motivation in searching for technological solutions to foster sustainable water supply, keeping the water–energy nexus and environmental impact in context. Membrane-based water treatment technologies outweigh all other conventional and competitive methods in terms of energy footprint, no chemical regenerants necessary, requirement of less space, possibility of continuous operation, environmental friendliness, scalability and product water quality. This involves application of pressure-driven membranes, e.g. ultrafiltration (UF), nanofiltration and reverse osmosis or a combination thereof based on the nature of industrial effluents in context. To reduce the energy demand of a particular or hybrid water treatment process, it is therefore proposed that future research on a membrane-based waste water treatment system should focus on membranes with high solvent throughput and selectivity towards the targeted contaminants, and fouling resistivity.

The polymeric UF membranes made of polysulfone (Psf), polyethersulfone (PES) and polyvinylidene fluoride (PVDF) undergo persistent deterioration of permeability and selectivity by fouling, which is an impact of a prolonged exposure to influent raw water streams [1–3]. These polymeric membranes are widely used in filtration industries owing to their good mechanical, thermal and chemical stabilities as well as amendable morphologies. However, their vulnerability to fouling owing to their inherent hydrophobic nature impedes their long-term sustainable performance [4]. The fouling in membrane systems is classified as crystalline fouling, organic fouling, microbial fouling, particulate fouling and colloidal fouling [5]. A gelatinous biofilm grows on the membranes at the upstream side with usage and, as a result, hydraulic resistance increases because of pore blockage and formation of additional cake layers. This effect results in the decline of the permeate flux and/or rise in the transmembrane pressure (TMP) during cross-flow filtration [6]. Therefore, it is always desirable to achieve a constant production rate by regaining the physico-chemical efficacy of the membranes through intense chemical or physical treatments. The factors responsible for membrane fouling not only reduce the lifetime of UF membranes but also increase energy consumption and ecological footprints by releasing chemical wastes from the unavoidable cleaning process into the environment and thus, finally, the operational cost of the membrane.

There are several strategies to circumvent these issues by either minimizing or mitigating the susceptibility of UF membranes to fouling. This is achieved by amending the structural features of the base polymers and/or impregnating various nanostructured materials within the polymeric matrix to tune the physico-chemical characteristics of the membrane. The surface modifications of membranes have been achieved through chemical modifications by different routes such as low-temperature plasma-induced graft polymerization [7], UV-initiated polymerization [8], γ-ray-induced graft copolymerization [9], electron beam irradiation [10], etc. Metal oxide nanoparticles such as Al_2_O_3_ [11], SiO_2_ [12], TiO_2_ [13], ZrO_2_ [14] and Fe_3_O_4_ [15], and tunable carbonaceous nanomaterials such as oxidized CNTs [16] and GO [17] have been incorporated into the porous polymeric matrix to achieve better antifouling properties.

Different facile strategies of surface modifications with manipulation of surface hydrophilicity, surface charge and surface roughness have been attempted using amphiphilic surface-modifying macromolecules (SMMs) for development of superior antifouling membranes [18]. The SMM was blended into a suitable base polymer for enhanced membrane properties, making use of the concept of ‘surface segregation’ in polymer science. In one such attempt, the surface modification of PVDF membranes was carried out using a fluorinated SMM additive [19], wherein the surface hydrophobicity of the membrane was increased on migration of the SMM to the surface that led to decrease in water permeability with increase in the content of the SMM. Novel fluorine-containing polyurethane SMMs have also been synthesized and blended with segmented polyurethanes to tune the surface chemistry of the polyurethane without affecting its bulk phase much [20]. In another study, the anti-biofouling property of the membrane was enhanced through enrichment of silver at the membrane surface by blending silver-containing SMMs into the host polymer matrix of the membrane [21]. Tailor-made charged SMMs were also developed to use as charge-enhancing additives for the preparation of PES-based UF membranes [22].

The research interest in the surface modification of membranes using TiO_2_ nanoparticles has grown significantly. In most of the cases, the striking photocatalytic activity of TiO_2_ nanoparticles has been explored to improve the quality of product water [23]. In a recent attempt [24], TiO_2_ nanoparticles of different size and shape were impregnated in the PVDF matrix using the NIPS method to substantiate the role of small-sized nanoparticles in obtaining better antifouling activities in the resultant UF membranes. In another study carried out by Yang *et al*. [25], it was demonstrated that a progressive incorporation of TiO_2_ nanoparticles in the casting Psf solution results in the change of the rheological properties such as from Newtonian to non-Newtonian viscous behaviour, which results in the alteration of membrane morphology and the deterioration of the UF membrane performance beyond a threshold concentration. Wu *et al*. [26] and Li *et al*. [27] correlated the effect of nanoparticle concentration with physico-chemical and macroscopical features of PES-TiO_2_-based UF membranes. A comparative investigation of TiO_2_-entrapped PES membranes and self-assembled TiO_2_-coated membranes with and without UV-irradiation [28] revealed the advantageous effect of irradiated membranes over the non-irradiated membranes in achieving higher fluxes and better antifouling properties. The hydrophilic nature of PES-based UF membranes was altered by a self-assembly of the hydroxyl groups of TiO_2_ nanoparticles, the sulfone groups and ether bond of PES through coordination as well as hydrogen bond interaction [29]. This study subsequently achieved a better performance and antifouling ability of UF membranes. Bae *et al*. [30] further investigated the fouling resistance of the TiO_2_ nanoparticles by preparing UF membranes via electrostatic self-assembly of TiO_2_ nanoparticles and the covalently tethered sulfonic acid groups of sulfonated PES.

The colloidal metal oxide particles of the nanosize regime undergo Brownian diffusion and thus are subject to homoaggregation owing to their attractive short-range thermodynamic interactions. This results in the formation of clusters or even aggregates, which consequently lead to an unstable dispersion [31]. Thus, in order to enhance the dispersion stability by preventing agglomeration, it is required to induce electro-steric repulsive forces through attachment of suitable organic ligands [32]. The coordinatively unsaturated Ti atoms of the TiO_2_ nanoparticles are reactive enough to reconstruct the vulnerable surfaces via functionalization by the suitable electron-donating organic ligands [33,34]. Jankovic *et al*. [35] determined the efficacy of several bidentate benzene derivatives in modulating the surface chemistry of the TiO_2_ nanoparticles.

In this study, we attempted to obtain superior physico-chemical features of the membrane matrix through impregnating the surface-tailored nano-TiO_2_ above and beyond the pristine nanoparticles. The objective was to explore the functional advantages of novel organofunctionalized nano-TiO_2_ and develop mixed-matrix membranes (MMMs) with better UF attributes. The facile functionalization of nano-TiO_2_ was obtained by incorporating two different organoligands, i.e. Tiron and chromotropic acid. The organofunctionalized nanoadditives were characterized using X-ray diffraction (XRD) analysis, Raman and Fourier transform infrared (FTIR) spectroscopy as well as X-ray photoelectron spectroscopy (XPS) to investigate the nature and extent of chemical functionalization. The MMMs were developed by the NIPS technique using the organoligand-tethered nanoadditives. The physico-chemical features of the MMMs were evaluated to delineate the role of each organoligand in modifying the properties of Psf-based UF membranes. This study also compared the performance of MMMs obtained with functionalized and pristine nano-TiO_2_. The variations in the UF performance and antifouling activity of the membranes were addressed to justify our approach towards superior industrial applications.

## 2. Experimental section

### 2.1. Materials

The base polymer (Psf—Mw: 30 kDa) was obtained from Solvay Specialties India Pvt. Ltd, Mumbai, India. The solvent, N-methyl-2-pyrrolidone (NMP) with minimum assay of 99.5% and porogen, polyvinyl pyrrolidone (PVP, K-30; Mw: 40 kDa) of AR grade (used without further purification) were procured from SRL Pvt. Ltd (Mumbai, India). The nanoparticles, anatase TiO_2_, denoted as nano-TiO_2_ (particle size: less than 25 nm, assay: 99.7% trace metal basis and specific surface area: 45–55 m^2^ g^−1^) were procured from Aldrich. For surface modification of the nano-TiO_2_, two different reagents, namely Tiron (4,5-dihydroxy-1,3-benzenedisulfonic acid disodium salt monohydrate, complexometric indicator grade) and chromotropic acid (1,8-dihydroxynaphthalene-3,6-disulfonic acid disodium salt dihydrate, technical grade), were procured from Fluka and Sigma-Aldrich, respectively. For the evaluation of the membrane rejection behaviour towards organic solutes, poly(ethylene glycol) (PEG, Mw: 35 kDa) and poly(ethylene oxide) (PEO, Mw: 100 kDa) were procured from Sigma-Aldrich. Bovine serum albumin (BSA) was procured from SRL Pvt. Ltd (Mumbai, India). The conductivity of Milli-Q ultra-pure water used in the experiments was below 2 µS cm^−1^.

### 2.2. Procedures for development of mixed-matrix membranes

#### 2.2.1. Synthetic routes adopted for surface modification of nano-TiO_2_

**Figure 1.**
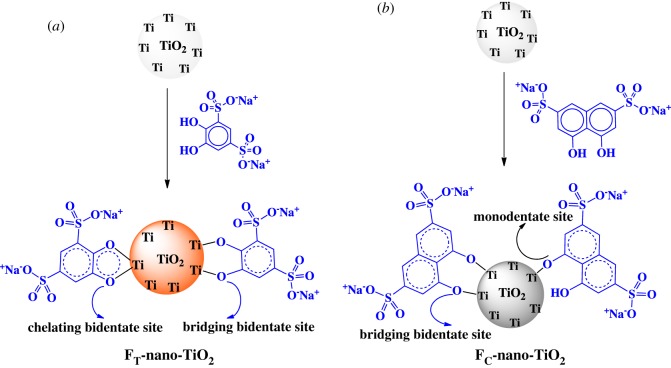
Schematics of functionalizations of anatase nano-TiO_2_ by Tiron and chromotropic acid; with proposed structures for (*a*) dark orange coloured FT-nano-TiO_2_ and (*b*) pale grey coloured FC-nano-TiO_2_, respectively.

The surface modifications of the anatase nano-TiO_2_ were made by two different facile synthetic routes. In the first approach (figure 1, pathway *a*), Tiron, comprising different binary functionalities (two adjacent reactive ─OH sites and two $-{\text{SO}}_{3}^{-}{\text{Na}}^{+}-{\text{SO}}_{3}^{-}{\text{Na}}^{+}$ $-{\text{SO}}_{3}^{-}\text{N}{\text{a}}^{+}-{\text{SO}}_{3}^{-}\text{N}{\text{a}}^{+}$ sites), was used to functionalize nano-TiO_2_ following a chemisorption procedure [36]. The nanoparticles were initially suspended into an aqueous solution of pH 2 (adjusted using HCl) under ultrasonic treatment for 2 h and then a freshly prepared aqueous solution of Tiron (4 mg ml^−1^, pH 2) was poured in. The suspension was subjected to vigorous stirring at room temperature for 30 min to allow the complete chemisorption of the catecholic compound onto the peripheral reactive sites of TiO_2_ nanoparticles. The acidic pH of the reaction medium was maintained to prevent the oxidation of the catecholic end groups of Tiron. The sulfonated nanoparticles were then rinsed with Milli-Q water until neutral pH was obtained and then centrifuged. The as-synthesized organofunctionalized nano-TiO_2_, denoted as F_T_-nano-TiO_2,_ was dried at 80°C for 24 h and stored for further usage.

The other route of the synthesis of organofunctionalized nano-TiO_2_ was followed as per the method (figure 1, pathway *b*) described by Ramesh *et al*. [37]. In this approach, nano-TiO_2_ (3 g) was added to 50 ml of aqueous solution containing 0.8 mg l^−1^ of chromotropic acid, comprising different binary functionalities (two reactive ─OH sites and two $-{\text{SO}}_{3}^{-}\text{N}{\text{a}}^{+}$ sites). The heterogeneous (inorganic–organic) mixture was stirred at room temperature for 2 h. The functionalized nano-TiO_2_ was then filtered off, washed with Milli-Q water several times and then centrifuged. The as-synthesized organofunctionalized nano-TiO_2_, denoted as F_C_-nano-TiO_2,_ was dried at 80°C for 24 h and stored for further usage.

#### 2.2.2. Preparation of polymer dope solutions

For the fabrication of mixed-matrix (Psf: F_T_/F_C_-nano-TiO_2_) membranes in sheet configurations, two different sets of polymer dope solutions (Set-A and Set-B), each having two different compositions, were prepared by incorporating different proportions of F_T_/F_C_-nano-TiO_2_ in hermetically sealed glass bottles. The amount of the nanoadditive, F_T_-nano-TiO_2_, was varied as 1 and 3 (w/w_Psf_)% in the dope solutions of Set-A (F_T_/1-nano-TiO_2_ and F_T_/3-nano-TiO_2_, respectively), comprising 20 (w/v_NMP_)% of Psf and 40 (w/w_Psf_)% of porogen, PVP. The amount of F_C_-nano-TiO_2_ was also varied as 1 and 3 (w/w_Psf_)% for the preparation of the dope solutions of Set-B (F_C_/1-nano-TiO_2_ and F_C_/3-nano-TiO_2_, respectively) with the amounts of PVP and Psf as in the case of Set-A. The dispersion of F_T_/F_C_-nano-TiO_2_ in NMP was subjected to ultrasonic treatment for 20 min, prior to the addition and subsequent mixing of the dried Psf beads and PVP, maintaining the specified compositions. The dope solutions were then vigorously agitated for several hours to achieve the complete dissolution of Psf and PVP in the solvent with homogeneously dispersed F_T_/F_C_-nano-TiO_2_. To get a comparative account, a set of polymer dope solutions (Set-C), including two dope solutions comprising nano-TiO_2_ at 1 and 3 (w/w_Psf_)% and one dope solution (Control) devoid of any nanoadditive, were further prepared following the aforementioned composition of polymer and porogen as well as the methodology. Then, the resultant viscous dope solutions (Set-A, Set-B, Set-C and Control, described in table 1) were kept overnight in a controlled atmosphere with temperature at 25 ± 1°C and the relative humidity at 35–40%, to eliminate the trapped air bubbles from the solutions.

**Table 1.** Specifications of precursor dope compositions and, respectively, derived membranes.

		Psf in NMP	PVP	F_T_-nano-TiO_2_	F_C_-nano-TiO_2_	nano-TiO_2_
UF membrane codes	dope codes	(w/v_NMP_)%	(w/w_Psf_)%	(w/w_Psf_)%	(w/w_Psf_)%	(w/w_Psf_)%
Psf: F_T_/1-nano-TiO_2_	Set-A	20	40	1	—	—
Psf: F_T_/3-nano-TiO_2_				3	—	—
Psf: F_C_/1-nano-TiO_2_	Set-B			—	1	—
Psf: F_C_/3-nano-TiO_2_				—	3	—
Psf: 1-nano-TiO_2_	Set-C			—	—	1
Psf: 3-nano-TiO_2_				—	—	3
Control-Psf	Control			—	—	—

#### 2.2.3. Preparation of mixed-matrix membranes

Prior to the fabrication of the MMMs of two different classes along with the control sample, cleaned glass plates (without having any fabric base) were taped at their parallel ends in such a way that each resulting membrane layer could achieve an estimated thickness of 200 µm. The as-prepared stable dope solutions of Set-A, Set-B, Set-C and the Control were manually cast onto the taped glass plates at a steady casting shear with the help of a well-dried ultra-smoothed glass roller. The entire assemblies with the cast films were immediately immersed into a precipitation bath containing ultra-pure water as the non-solvent, maintained at room temperature, for immersion precipitation. To ensure adequate exchange between the solvent and the non-solvent followed by the complete removal of porogen and solvent from the membrane matrices, the prepared membranes were taken out of the water bath and rinsed several times with water. The entire casting process was carried out in a controlled atmosphere, where temperature and relative humidity were maintained at 25 ± 1°C and 35–40%, respectively. The membranes, developed under the invariable casting condition, were properly stored in a water bath. The membranes were categorized on the basis of the difference in the specified compositions and were accordingly designated as Psf: F_T_/1-nano-TiO_2_ UF and Psf: F_T_/3-nano-TiO_2_ UF for the dope solutions of Set-A; Psf: F_C_/1-nano-TiO_2_ UF and Psf: F_C_/3-nano-TiO_2_ UF for the dope solutions of Set-B; Psf: 1-nano-TiO_2_ UF and Psf: 3-nano-TiO_2_ UF for the dope solutions of Set-C; and the Control-Psf UF (table 1).

### 2.3. Characterization of as-synthesized organofunctionalized nano-TiO_2_

The phase purity and structure of the functionalized derivatives of nano-TiO_2_ (F_T_/F_C_-nano-TiO_2_) were determined by X-ray powder diffraction (XRD) analysis. The data were collected on a Philips X'Pert pro X-ray diffractometer using Cu-Kα radiation (*λ* = 1.5418 Å) at 40 kV and 30 mA. The average crystallite size was estimated using the Scherrer equation (*t *= *Kλ*/*B* cos *θ*, where *t = *average crystallite size in Å, *K =* Scherrer constant, usually taken as 0.9 Å, *λ *= X-ray wavelength, *θ* is the Bragg angle and *B *= integral breadth of a reflection located at 2*θ*). The spontaneous Raman spectra of F_T_/F_C_-nano-TiO_2_ were obtained using an STR–300 micro-Raman spectrometer (SEKI Technotron, Japan). The data were acquired over a spectral range of 50–1000 cm^–1^ at room temperature for an identical acquisition period of 200 s. The samples were excited at 532 nm (power approx. 20 mW at the sample spot, diode-pumped solid-state laser) using a 10× objective lens (Olympus). The scattered light was collected by the same objective lens and a fibre-coupled 300 mm spectrograph (Acton series SP 2300i, 1200 g mm^−1^) and detected by a thermo-electric cooled (−75°C) charge-coupled device. The FTIR spectra of the two different classes of F_T_/F_C_-nano-TiO_2_ were recorded in the attenuated total reflectance (ATR) mode using a Bruker Vertex 70 FTIR spectrometer operating in a range of 400–4000 cm^−1^. For X-ray photoelectron spectroscopic (XPS) technique, a DESA–150 electron analyser (Staib Instruments, Germany) equipped with a Mg-Kα X-ray source (1253.6 eV) was employed for characterization purposes. The binding energy scale of the spectrometer was calibrated with the Au–4f_7/2_ photo-peak at a binding energy (B.E.) of 83.95 eV. The spectra were recorded as intensity (number of counts per second) versus B.E. The multiplex photo-peaks were subjected to the Gaussian functions to fit the curves, and then the peak area as well as the full width at half maximum (FWHM) were determined.

### 2.4. Physico-chemical characterizations of mixed-matrix membranes

The cross-sectional morphology of the MMMs was investigated using a field emission scanning electron microscope (FE-SEM, Model: AURIGA, Carl Zeiss, Germany). For the purpose of imaging, a piece (0.5 cm^2^) of the membranes was cut and fractured into smaller-sized strips in liquid nitrogen. Then, the cross-sectional layers were sputter coated with Au/Pd (60/40) using a sputter coater (Model No. K550X Emitech), under the optimum conditions (sputtering time: 60 s, sputter current: 30 mA and tooling factor: 2.3), in order to reduce the effect of electrostatic charging on the electrically non-conductive membrane samples. All the micrographs were recorded at magnifications of 1750× and 20 000× at an identical acceleration voltage of 5 kV, while operating in the secondary electron mode.

The quantitative elemental analysis of the selective membrane surfaces was simultaneously performed by using an energy dispersive X-ray spectrometer (EDX, INCA, Oxford Instruments, UK), coupled to the SEM and a microanalysis system. The microanalysis system was equipped with an ultrathin beryllium window and a Si-detector (20 mm^2^). In the EDX analysis, an acceleration voltage of 20 kV and magnification of 4000× were used. The spectra were acquired for the elements of interest, i.e. Ti of the organofunctionalized nano-TiO_2_, impregnated in the membrane matrices at varying concentrations, and C, S and O.

The skin surface topography of the membranes was characterized by using an atomic force microscope (AFM, Model: SOLVER next, NT-MDT, Russia). The membranes were excised into square pieces of approximately 1 cm^2^ and pasted onto a metal substrate. The rectangular cantilever NSG 10 (NT-MDT, Russia) made of Si_3_N_4_ with a spring constant of 11.8 N m^−1^, a typical resonance frequency of 240 kHz, and a nominal tip apex radius of 10 nm with a high aspect ratio, was used for the purpose of scanning. The scanning was done in the semi-contact mode on a 20 × 20 µm area of the membrane in air and at room temperature with a scanning frequency of 0.1 Hz. The scanned regions were flattened using a second-order polynomial to remove the curvatures and slopes from the image and then the resulting best fit was subtracted from it. The surface roughness parameter of the membranes, evaluated by the NOVA-P9 software, was taken as the height profile of the images in terms of average roughness (*R*_a_) and root mean square roughness (*R*_q_).

The static sessile drop method was adopted to determine the contact angles, and thereby analyse the overall surface hydrophilic features of the membranes having physico-chemical heterogeneities. A drop shape analyser (DSA 100 of KRÜSS Gmbh, Germany) equipped with the DSA 1 v. 1.92 software was used to measure the water contact angle. A 3 µl drop of the probe solvent (water) was deposited using a microsyringe needle on the membrane surface. The equilibrium contact angle values were measured at the membrane–solvent–air interface with an equal residence time of 60 s. The mean contact angle was calculated by carrying out such measurements at eight different locations for each membrane surface.

The electrokinetic characteristics of the membranes were evaluated by a ZetaCAD electrokinetic analyser (CAD Inst., France), which consisted of a quartz cell configuration that holds two flat sheets of each membrane in such a way that the probed membranes remained separated by spacers and their skin layers facing each other create a slit channel for the tangential flow of electrolytic solution across the membrane. The streaming potential, generated by the bidirectional flow of 10^−3^ M KCl as the background electrolyte solution, under applied pressure gradient across the membrane, was measured by Ag/AgCl electrodes, equipped with the cell. The zeta potential (*ζ)* of the membranes was evaluated by the streaming potential values using the Helmholtz–Smoluchowski equation as follows:

2.1 $${\text{V}}_{\mathrm{st}}=\frac{\varepsilon }{\lambda \eta }\zeta ,$$

where *ε* is the dielectric constant (*ε *= *ε*_0_*ε*_r_, being *ε*_r_ the relative dielectric constant and *ε*_0_ the vacuum permittivity), and *η* and *λ* are the viscosity and conductivity of the electrolyte medium, respectively. An average value of *ζ* was derived from three replicates and the measurement error was within ±0.8 mV.

The porosity of the membranes was measured by the gravimetric method. A circular piece of each membrane with an area *A* and thickness *h* was weighed after taking it out of the distilled water bath and then carefully removing the superficial water with filter paper. The wet membranes were dried in a vacuum oven at 75°C for 24 h before measuring the weight in the dry state. From the weights of the membrane samples in wet (*W*_0_) and dry (*W*_1_) states, the porosity (*ø*) of each membrane was calculated using the following equation:

2.2 $$\phi (\%)=\frac{W_{0}-W_{1}}{\rho_{w}Ah}\times 100,$$

where *ρ*_w_ is the density of pure water at room temperature. To minimize the experimental error, the measurements were carried out in duplicate and an average value was considered. On the basis of the porosity of the membranes, the mean pore radius (*r*_m_ in nanometres) was determined by the Guerout–Elford–Ferry equation [38] as follows:

2.3 $$r_{m}=\sqrt{\frac{(2.9-1.75\phi )\times 8\eta hv}{\phi \times A\times \Delta P}},$$

where *ϕ* (%) and *h* (m) denote the porosity and thickness of the membrane, respectively, and the viscosity of water (8.9 × 10^−4^ Pa s) is represented as *η*. The volume of water that permeated per unit of time (*v* in m^3 ^s^−1^) is considered to pass through an effective membrane area of *A* (square metres), under 1 bar of TMP (Δ*P*).

### 2.5. Evaluation of ultrafiltration performances of mixed-matrix membranes

The efficiency of molecular separation was evaluated by analysing the rejection behaviour of the membranes towards neutral organic solutes, such as PEG with an average Mw of 35 kDa and PEO with an average Mw of 100 kDa. The test solutions were prepared by dissolving PEG and PEO in ultra-pure water at a concentration of 200 ppm. The membranes with an identical effective area of 14.5 cm^2^ were employed in a cross-flow filtration unit, being operated under a TMP of 1 bar at room temperature. The measurements were repeated with three different coupons of each membrane and the average values were considered. The concentrations of PEG and PEO in both the feed and product solutions were measured by analysing the total organic carbon (TOC) content of the samples using a TOC analyser (ANATOC-II, SGE Analytical Science, Australia). The percentage rejection of the probe organic solutes (*R*_PEG/PEO_) was determined using the following equation:

2.4 $$R_{\mathrm{Solute}}=\frac{C_{F}-C_{P}}{C_{F}}\times 100\%,$$

where *C*_P_ and *C*_F_ are permeate and feed concentrations, respectively.

The steady-state solvent flux (*J* in l m^−2^ d^−1^ or LMD) was determined by direct and replicate measurements of the permeate flow, i.e. the volume of permeate (*V*, in litres) collected during the time (*T*, in days) through the membrane area (*A*, in square metres) and considering the following equation. Prior to all UF test experiments, the membranes were subjected to a hydraulic compaction for 1 h in water at the standard UF test conditions to achieve stabilized performances of the membranes. The pure water flux (*J*_o_) was measured at 1 bar of TMP.

2.5 $$J=\frac{V}{AT}.$$

Thereafter, a protein solution of BSA (1000 ppm) in phosphate buffer (pH 7.4) was allowed to permeate through the membranes in the dead-end filtration mode at the same TMP for 30 min and the respective flux (*J*_1_) was estimated. The concentration of BSA in the feed and permeate solutions was analysed by a TOC analyser, and the respective percentage rejection of BSA (*R*_BSA_) was calculated by equation (2.4). After filtration of the protein solution, the membranes were back-washed with ultra-pure water for 30 min at a similar TMP and subsequently the water flux (*J*_2_) of the cleaned membrane was measured. The antifouling property of the membranes was evaluated by determining the flux recovery ratio (FRR) [39], which was calculated using the following equation:

2.6 $$\text{FRR}=(J_{2/}J_{0})\times 100\%.$$

The flux decline caused by the reversible and irreversible protein fouling, designated by *R*_r_ and *R*_ir_, was defined by the following equations, respectively [39]:

2.7 $$R_{r}=\frac{J_{2}-J_{1}}{J_{0}}\times 100\%$$

and

2.8 $$R_{\mathrm{ir}}=\frac{J_{0}-J_{2}}{J_{0}}\times 100\%.$$

The overall membrane fouling was considered as a collective outcome of the reversible and irreversible fouling, thus the degree of flux decline caused by the overall protein fouling (*R*_t_) in the process of UF was defined by the following equation:

2.9 $$R_{t}=R_{r}+R_{\mathrm{ir}}=\left(1-\frac{J_{1}}{J_{0}}\right)\times 100\%.$$

## 3. Results and discussions

### 3.1. Analysis of physico-chemical characteristics of organofunctionalized nano-TiO_2_

**Figure 2.**
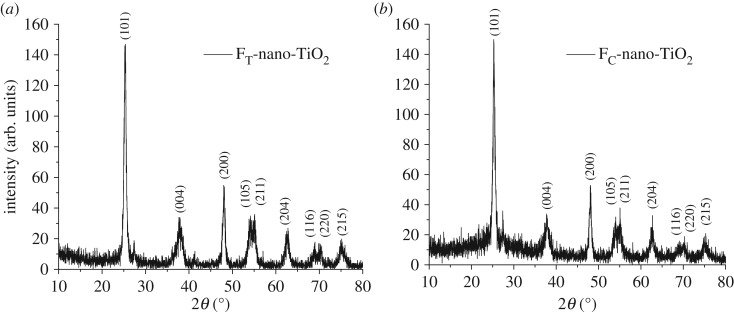
XRD patterns of organofunctionalized nano-TiO_2_, (*a*) FT-nano-TiO_2_ and (*b*) FC-nano-TiO_2_.

The XRD patterns of the synthesized derivatives of nano-TiO_2_, i.e. F_T_-nano-TiO_2_ and F_C_-nano-TiO_2_, are shown in figure 2*a* and *b*, respectively. The XRD pattern depicted in figure 2*a* reveals the presence of a strong diffraction peak at 25.3° (FWHM: 0.6561), indexed to (101) plane diffraction and a few successive peaks with lower intensities at 37.8°, 48.1°, 54.2°, 55.2°, 62.6°, 68.9°, 70.1° and 75.1°, which are indexed to the (004), (200), (105), (211), (204), (116), (220) and (215) plane diffractions, respectively, and can accordingly be attributed to the anatase phase of F_T_-nano-TiO_2_. In the XRD pattern shown in figure 2*b*, an intense diffraction peak appears at 25.3° (FWHM: 0.7071) followed by a few peaks of lower intensities appearing at 37.9°, 48.1°, 54.3°, 55.1°, 62.6°, 68.8°, 70.3° and 75.2° that are indexed to the (101) and (004), (200), (105), (211), (204), (116), (220) and (215) plane diffractions, respectively, and can be attributed to the anatase phase of F_C_-nano-TiO_2_ [40]. The most abundant and thermodynamically stable low-energy (101) facets of anatase nano-TiO_2_ are supposed to be the reactive surfaces, wherein the facile chemisorptions occur when two potentially labile protons—the one associated with the catecholic oxygens in Tiron and the other of the hydroxyl groups in chromotropic acid—of each organoligand induce chemical interactions with the chelating surface Ti atoms of the nano-TiO_2_ [41]. In accordance with the Scherrer formula, the average crystallite sizes of F_T_-nano-TiO_2_ and F_C_-nano-TiO_2_ were estimated to be 12.97 nm and 12.03 nm, respectively.

**Figure 3.**
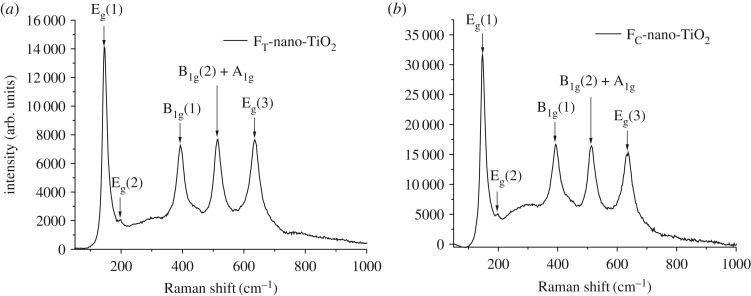
Raman spectra of organofunctionalized nano-TiO_2_, (*a*) FT-nano-TiO_2_ and (*b*) FC-nano-TiO_2_ at room temperature.

The Raman spectra of the F_T_-nano-TiO_2_ and F_C_-nano-TiO_2_, acquired at room temperature, are shown in figures 3*a* and *b*, respectively. The anatase phase of nano-TiO_2_ is known to belong to a space group D^19^_4 h_, *I*4_1_/amd with two primitive unit cells, each containing two units of the nano-TiO_2_ [42]. The factor group analysis revealed that there are six Raman active vibrations: A_1 g_+2B_1 g_+3E_g_. The characteristic frequencies of the Raman bands as observed in figure 3*a* and *b* are 145.2, 198.2, 394.2, 515.8, 634.9 cm^−1^ and 148.3, 197.6, 394.2, 514.4, 637.4 cm^−1^, respectively. Among these, the bands with highest intensities at 145.2 cm^−1^ and 148.3 cm^−1^, the bands with comparatively lower intensities at 634.9 cm^−1^ and 637.4 cm^−1^, and the bands with very low intensities at 198.2 cm^−1^ and 197.6 cm^−1^ are assigned to the E_g_ modes of F_T_-nano-TiO_2_ and F_C_-nano-TiO_2_, respectively. Both of the bands appearing at 394.2 cm^−1^ are referred to the B_1 g_ mode. The higher-frequency bands, at 515.8 and 514.4 cm^−1^, are the doublet of the A_1 g_ and B_1 g_ modes.

**Figure 4.**
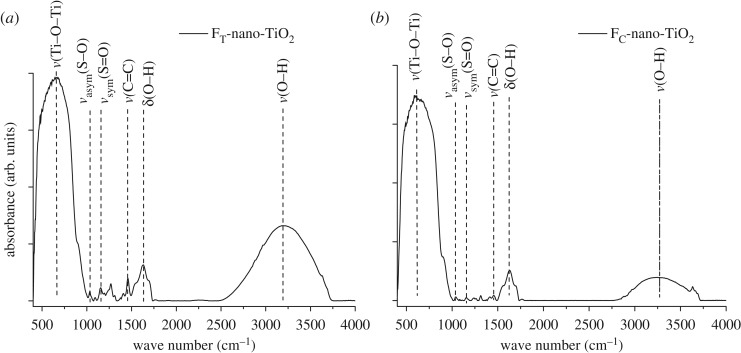
FTIR spectra of organofunctionalized nano-TiO_2_, (*a*) FT-nano-TiO_2_ and (*b*) FC-nano-TiO_2_.

The FTIR spectra (figure 4*a* and *b*) confirm the functionalization of nano-TiO_2_ using the organoligands and the formation of F_T_-nano-TiO_2_ and F_C_-nano-TiO_2_. A high-intensity band attributed to the characteristic Ti─O─Ti stretching vibration appears at 638 cm^−1^ for F_T_-nano-TiO_2_ and at 634 cm^−1^ for F_C_-nano-TiO_2_. The bands at 1034 and 1038 cm^−1^ can be assigned to the asymmetric stretching vibration of S─O groups, ν_asym_(S─O). The bands at 1155, 1158 and 1245, 1240 cm^−1^ correspond to the symmetric and asymmetric stretching vibrations of the S═O groups, i.e. ν_sym_(S═O) and ν_asym_(S═O), respectively, which substantiate the facile chemisorptions of Tiron and Chromotopic acid onto the reactive facets of nano-TiO_2_ [43]. The bands at 1458 and 1460 cm^−1^ are ascribed to the characteristic stretching vibrations of the aromatic rings, ν(C═C) originated from the benzene and naphthalene rings of the F_T_-nano-TiO_2_ and F_C_-nano-TiO_2_, respectively [35]. The broad bands at 3200 and 3263 cm^−1^, and comparatively narrower bands at 1630 and 1632 cm^−1^ are attributable to the stretching and scissoring vibrations of the O─H groups, ν(O─H) and δ(O─H), originating from the adsorbed water molecules and hydroxyl groups on the surface of F_T_-nano-TiO_2_ and F_C_-nano-TiO_2_, respectively. Moreover, a close comparison of the intensities of ν(O─H) revealed that the hydroxyl groups responsible for the surface hydrophilic sites are more abundant in F_T_-nano-TiO_2_ than in F_C_-nano-TiO_2_. This seems to be an outcome of the more facile chemisorption of Tiron over chromotropic acid owing to their structural differences.

**Figure 5.**
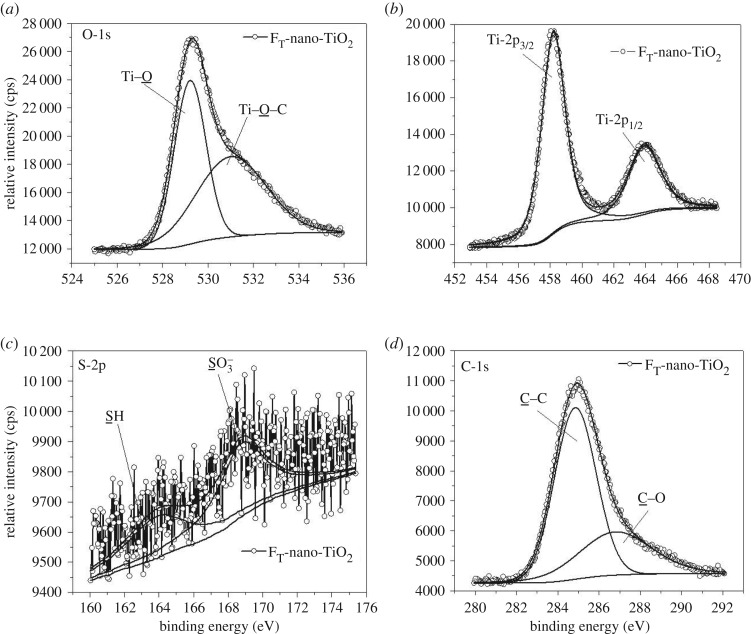
High resolution photoelectron spectra obtained from FT-nano-TiO_2_ (line with bullets: experimental data; solid line: curve fit of the experimental data); (*a*) O-1s, (*b*) Ti-2p, (*c*) S-2p and (*d*) C-1s.

**Figure 6.**
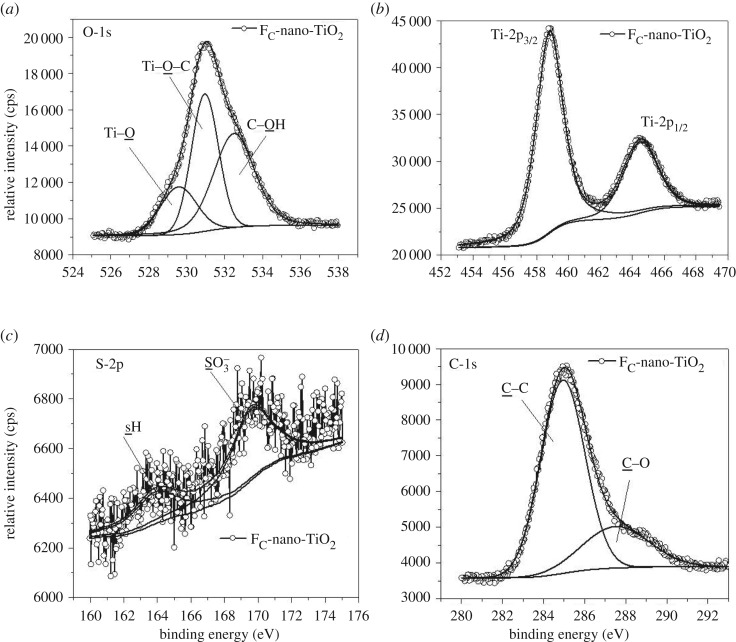
High resolution photoelectron spectra obtained from FC-nano-TiO_2_ (line with bullets: experimental data; solid line: curve fit of the experimental data); (*a*) O-1s, (*b*) Ti-2p, (*c*) S-2p and (*d*) C-1s.

The detailed mechanisms of chemisorptions of Tiron and chromotropic acid onto the surfaces of nano-TiO_2_ were further explored by XPS analysis. The variations in B.E. and respective peak areas (table 2) acquired from the deconvoluted core-level X-ray photoelectron spectra of O-1s (figures 5*a* and 6*a*), Ti-2p (figures 5*b* and 6*b*), S-2p (figures 5*c* and 6*c*) and C-1s (figures 5*d* and 6*d*) revealed that the structural heterogeneities of the organoligands modulate the molecular-level interactions in F_T_-nano-TiO_2_ and F_C_-nano-TiO_2_. The curve fitting and deconvolution of O-1s core-level spectra exhibited an asymmetric pattern for each spectrum (figure 5*a*), corresponding to F_T_-nano-TiO_2_, and yielded two constituent spectra. By contrast, the spectrum with more asymmetry (figure 6*a*), attributed to F_C_-nano-TiO_2_, constitutes three distinctive spectra. Thus, herein we propose that different states of chemical fixation exist in the probed organofunctionalized nanoparticles. The peaks located at a B.E. of 529.2 (FWHM: 1.65 eV) and 529.5 eV (FWHM: 2.24 eV) (table 2) could be attributed to the O-atoms (Ti−O−Ti) of F_T_-nano-TiO_2_ and F_C_-nano-TiO_2_, respectively. The broad peaks at a higher B.E. of 531.0 eV in both the cases, however, were associated with higher FWHM for the former (3.63 eV) rather than the latter (1.68 eV). This was assigned to the O-atoms of the precursor complexing ligands, bonded to the nano-TiO_2_ surface as C−O−Ti. This route of chemical fixation is associated with the occurrence of dissociative adsorptions onto the (101) facets, which are further supposed to occur when the covalently bonded labile protons dissociate from both the −OH groups of each of the organoligands, i.e. Tiron and chromotropic acid, and subsequently bridge with Ti atoms onto the surface of nano-TiO_2_, resulting in bidentate binuclear bridging during fixation (figure 1*a*,*b*) [35,44]. In the case of F_T_-nano-TiO_2_, the other possibility is a concurrent but competitive mode of chemisorption, where chelating mononuclear bidentate sites are formed through bonding of both the O-atoms of adjacent −OH groups (catecholate type) to a single Ti atom (figure 1*a*) [35,45]. The high intensity and relative area of the concerned peak (figure 5*a* and table 2) substantiate the contribution of the proposed geometry in mixed coverage of bridging and chelating structures in F_T_-nano-TiO_2_. The appearance of a third constituent peak in the deconvoluted spectrum of O-1s (figure 6*a*) revealed the concurrent presence of an additional geometry along with the aforementioned bridging bidentate one in F_C_-nano-TiO_2_ (schematically presented in figure 1*b*). The peak at a B.E. of 532.5 eV with FWHM of 2.54 eV is attributed to the O-atoms of −OH groups, which are covalently bonded to the ring C-atoms of chromotropic acid, and thus signifying the contribution of monodentate geometry in F_C_-nano-TiO_2_ [41]. It happens when one −OH group gets deprotonated and subsequently makes a bond with the Ti atom, while the neighbouring one remains as such. The bulkiness of chromotropic acid may be ascribed to the formation of monodentate geometry, instead of the chelating mononuclear bidentate geometry like in F_T_-nano-TiO_2_. The comparison of the relative peak areas implies that the monodentate geometric configuration is energetically more preferred than the bridging bidentate geometric configuration, and it is reasonable to see the occupancy of the former on the surface of F_C_-nano-TiO_2_ to be greater than the latter.

**Table 2.** The summary of curve fitting of different XPS peaks for F_T_-nano-TiO_2_ and F_C_-nano-TiO_2_.

	Ti-2p3/2 & Ti-2p1/2			S-2p			C-1s			O-1s		
codes	B.E. (eV)	FWHM (eV)	peak area (%)	B.E. (eV)	FWHM (eV)	peak area (%)	B.E. (eV)	FWHM (eV)	peak area (%)	B.E. (eV)	FWHM (eV)	peak area (%)
F_T_-nano-TiO_2_	458.0	1.74	67.98	163.8	4.21	43.08	284.4	2.38	67.50	529.2	1.65	47.19
	464.0	2.40	32.02	168.7	3.33	56.92	286.8	4.15	32.50	531.0	3.63	52.81
F_C _− nano-TiO_2_	458.8	1.99	70.29	163.8	3.40	34.30	284.8	2.53	75.42	529.5	2.24	17.70
	464.4	2.57	29.71	169.6	2.95	65.70	287.5	3.84	24.58	531.0	1.68	38.21
										532.5	2.54	44.09

The Ti-2p spectra, represented in figures 5*b* and 6*b*, exhibit the presence of strong peaks at 458.0 and 458.8 eV with FWHM of 1.74 and 1.99 eV attributed to Ti-2p_3/2_, and weak peaks at 464.0 and 464.4 eV with FWHM of 2.40 and 2.57 eV assigned to Ti-2p_1/2_, where the two peaks originate from the surface of Ti^4+^ atoms in the anatase phase of F_T_-nano-TiO_2_ and F_C_-nano-TiO_2_, respectively [41]. The distinct shifts of B.E. of Ti-2p_3/2_ and Ti-2p_1/2_, by 0.8 and 0.4 eV, respectively, with the concurrent broadening of the peaks substantiate that the chromotropic acid seems to be capable of strongly abstracting the electrons from the adjacent Ti^4+^ in the bridging structure of F_C_-nano-TiO_2_ when compared with the Tiron in F_T_-nano-TiO_2_. The component peaks are in the 2 : 1 ratio of Ti-2p_3/2_:Ti-2p_1/2_ peak intensity, but the ratio is slightly higher in the case of the former when compared with the latter. The deconvoluted core-level S-2p spectra, presented in figures 5*c* and 6*c*, exhibit two contributing peaks, wherein the more intense peaks appearing at 168.7 and 169.6 eV, with FWHM of 3.33 and 2.95 eV, respectively, are assigned to the sulfur of $-{\text{SO}}_{3}^{-}{\text{H}}^{+}$ groups attached to the benzene ring of the bridged Tiron and naphthalene ring of the bridged chromotropic acid. The less intense peaks at 163.8 eV (FWHM: 4.21 and 3.40 eV) for both correspond to the presence of sulfur in the converted −SH groups [46]. The greater electron-withdrawing ability of the naphthalene ring exerts a higher deshielding effect on the sulfur of the $-{\text{SO}}_{3}^{-}{\text{H}}^{+}$ groups in F_C_-nano-TiO_2_ and thereby causes a higher chemical shift than in the case of F_T_-nano-TiO_2_. The ratios of relative intensity (table 2) of the component peaks signify the relative conversion of the $-{\text{SO}}_{3}^{-}{\text{H}}^{+}$ sites into the −SH, which is more constrained in F_C_-nano-TiO_2_ than in F_T_-nano-TiO_2_. However, the conversion occurs without any intervention by an external reducing or hydrogenating agent, and thus could be attributed to the effect of photoelectron exposure during the XPS measurements [46]. The deconvolution of the C-1s core-level spectra of as-synthesized F_T_-nano-TiO_2_ and F_C_-nano-TiO_2_, presented in figures 5*d* and 6*d*, respectively, exhibits two distinct peaks for C atoms residing in different chemical environments. The intense component peaks appearing at the lowest B.E. of 284.4 and 284.8 eV (table 2), with respective FWHM of 2.58 and 2.53 eV, are ascribed to the non-oxygenated ring C of C─C or C─H [47]. The broader but less intense peaks appearing at B.E. of 286.8 and 287.5 eV, with FWHM of 4.15 and 3.84 eV, respectively, are referred to the C atoms of C─O segments [47,48]. The effect of deshielding in subsequent chemical shift values is more pronounced in F_C_-nano-TiO_2_ than F_T_-nano-TiO_2_, which is attributable to the difference in the structural attributes of the complexing organoligands.

### 3.2. Analysis of physico-chemical features of mixed-matrix membranes

**Figure 7.**
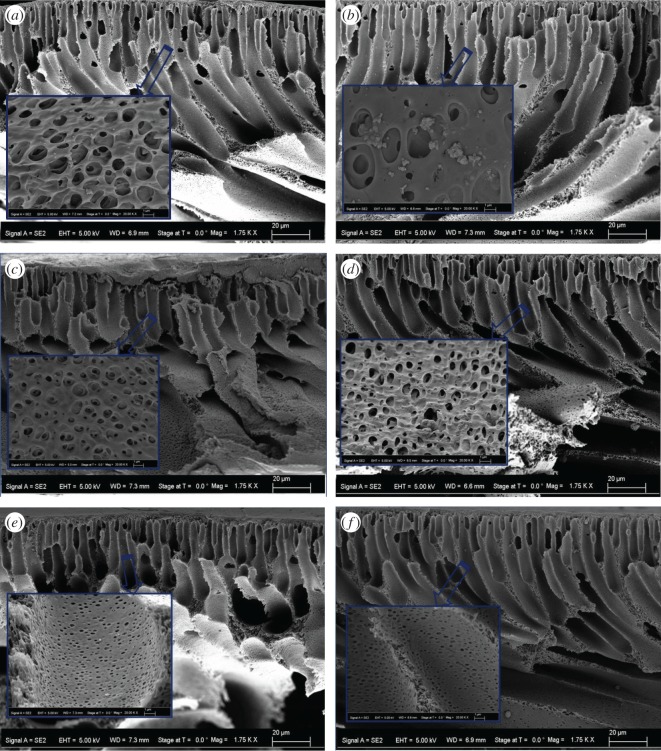
Characteristic cross-sectional morphology acquired by FE-SEM at the magnifications 1750x and 20000x (inset), for MMMs: (*a*) Psf: 1-nano-TiO_2_ UF and (*b*) Psf: 3-nano-TiO_2_ UF; (*c*) Psf: FT/1-nano-TiO_2_ UF and (*d*) Psf: FT/3-nano-TiO_2_ UF; (*e*) Psf: FC/1-nano-TiO_2_ UF and (*f*) Psf: FC/3-nano-TiO_2_ UF.

The cross-sectional morphologies of the MMMs acquired through FE-SEM are presented in figure 7. The pores are asymmetric in size, as those in the dense skin regions are smaller than those formed at the interior substructural regions. This can clearly be taken as a typical signature of the NIPS process [49]. The comparison of Psf: 1-nano-TiO_2_ UF (figure 7*a*) and Psf: 3-nano-TiO_2_ UF (figure 7*b*) suggests the formation of aggregation, which indicates the presence of clusters of nano-TiO_2_ within the porous substructural region of the latter membrane at a higher loading of the pristine nano-TiO_2_. The stability of the nanoparticle dispersion varies with the inter-particle interaction, depending on the van der Waals pair interaction energy [50]. This energy parameter is proportionally related to the Hamaker constant, which inversely depends on the inter-particle distances and varies with the concentration as well as surrounding chemical environment [31,50]. In the case of nano-TiO_2_, the inter-particle attractive force remains high during their dispersion in NMP, so they tend to come closer and induce flocculation with progressive concentration. This resulting effect is manifested through the presence of distinct nano-TiO_2_ clusters in Psf: 3-nano-TiO_2_ UF. However, the cross-sectional morphologies of Psf: F_T_-nano-TiO_2_ UF (figure 7*c* and *d*) and Psf: F_C_-nano-TiO_2_ UF (figure 7*e* and *f*) illustrate that there is no evidence of aggregation when organofunctionalized nano-TiO_2_, i.e. F_T_-nano-TiO_2_ and F_C_-nano-TiO_2_, are impregnated at similar higher concentrations. Such features stem from the tethering of the organoligands on the surfaces of nano-TiO_2_ that restricts their coagulation propensity in the dispersion medium by inducing steric as well as electrostatic repulsion, and in this way reduces the attractive van der Waals inter-particle potential [51].

**Figure 8.**
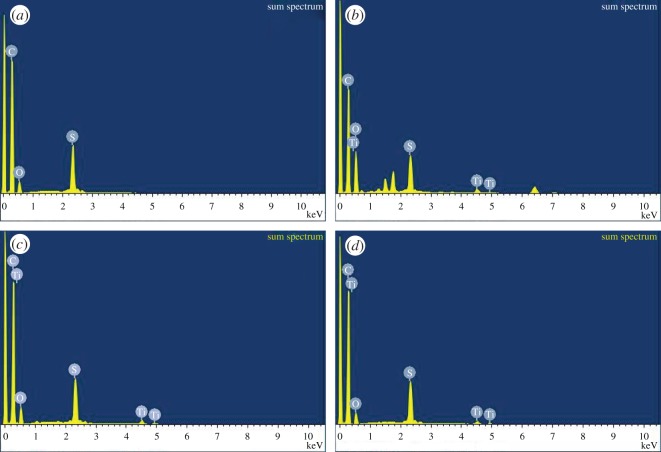
EDX spectra of skin regions of membranes: (*a*) Control-Psf UF, (*b*) Psf: 3-nano-TiO_2_ UF, (*c*) Psf: FT/3-nano-TiO_2_ UF and (*d*) Psf: FC/3-nano-TiO_2_ UF.

The EDX spectra of the representative membranes, namely Control-Psf UF, Psf: 3-nano-TiO_2_ UF, Psf: F_T_/3-nano-TiO_2_ UF and Psf: F_C_/3-nano-TiO_2_ UF, are presented in figure 8*a*,*b*,*c*,*d*, respectively. All the spectra exhibit the presence of C, S and O peaks, whereas the distinct and additional elemental peak of Ti is shown in the MMMs only. The variations in nanoparticle density, roughly within the skin regions, examined through the quantitative elemental analysis of the membranes in terms of the relative weight and atomic percentages, are presented in table 3. The contributions of C and S indicate that the differential distributions of these specific elements reduce sharply from the bare membrane to the MMM impregnated with nano-TiO_2_. However, there is a considerable increase in these elements when both organofunctionalized nano-TiO_2_ are impregnated at similar concentrations. The comparison of the relative weight and atomic percentages of O and Ti in the studied membranes indicates that the skin region of Psf: 3-nano-TiO_2_ UF is significantly populated with the aggregated or clustered nano-TiO_2_, and subsequently the surface chemistry gets severely altered in comparison to the skin region of Control-Psf UF. It is also observed that the nanoparticle densities, measured as weight and atomic percentages of Ti and O in the skin regions of Psf: F_T_/3-nano-TiO_2_ UF and Psf: F_C_/3-nano-TiO_2_ UF, are reduced. These indeed imply the segregated presence of the organofunctionalized nano-TiO_2_ within the skin regions of the respective MMMs. In general, functionalization thus ensures a facilitated distribution of both F_T_-nano-TiO_2_ and F_C_-nano-TiO_2_, over the pristine nano-TiO_2_ in the resultant MMMs.

**Table 3.** Elemental analysis of membrane skin layers: Control-Psf UF, Psf: 3-nano-TiO_2_ UF, Psf: F_T_/3-nano-TiO_2_ UF and Psf: F_C_/3-nano-TiO_2_ UF.

	elemental (C) quantification		elemental (O) quantification		elemental (S) quantification		elemental (Ti) quantification	
UF membrane codes	Wt (%)	atomic (%)	Wt (%)	atomic (%)	Wt (%)	atomic (%)	Wt (%)	atomic (%)
Control-Psf	79.83 ± 0.12	86.31	13.55 ± 0.11	11.00	6.62 ± 0.09	2.69	—	—
Psf: 3-nano-TiO_2_	60.22 ± 0.18	68.75	33.54 ± 0.21	28.75	5.05 ± 0.14	2.16	1.19 ± 0.15	0.34
Psf: F_T_/3-nano-TiO_2_	75.31 ± 0.27	82.66	17.77 ± 0.15	14.64	5.81 ± 0.18	2.39	1.11 ± 0.18	0.31
Psf: F_C_/3-nano-TiO_2_	79.68 ± 0.22	86.42	13.35 ± 0.17	10.87	6.08 ± 0.23	2.47	0.89 ± 0.26	0.24

The topographical features were investigated through AFM for the skin surfaces of selective membranes to probe the role of functionalization in modulating the effective distribution of nano-TiO_2_ within the polymeric host matrix. The roughness parameters, *R*_a_ and *R*_q_, presented in table 4, were found to increase significantly from Control-Psf UF (8.89 and 11.63 nm) to Psf: 1-nano-TiO_2_ UF (21.39 and 28.81 nm), indicating a concomitant increase in the effective surface area of the membranes upon the impregnation of the nanoadditive at a lower loading. However, both *R*_a_ and *R*_q_ values declined upon the impregnation of the organofunctionalized nanoadditives as these parameters were 17.81 and 25.07 nm and 9.46 and 17.94 nm, respectively, for F_T_-nano-TiO_2_ and F_C_-nano-TiO_2_. It was observed that the decline was more pronounced in the latter membrane sample, and this is in accordance with the enhanced dispersion and the subsequent facilitated distribution of the F_C_-nano-TiO_2_ when compared with the F_T_-nano-TiO_2_, as discussed earlier.

**Table 4.** Surface roughness parameters of membranes: Control-Psf UF, Psf: 1-nano-TiO_2_ UF, Psf: F_T_/1-nano-TiO_2_ UF and Psf: F_C_/1-nano-TiO_2_ UF.

	UF membrane codes			
roughness parameters (nm)	Control-Psf	Psf: 1-nano-TiO_2_	Psf: F_T_/1-nano-TiO_2_	Psf: F_C_/1-nano-TiO_2_
*R*_a_	8.89	21.39	17.81	9.46
*R*_q_	11.63	28.81	25.07	17.94

**Figure 9.**
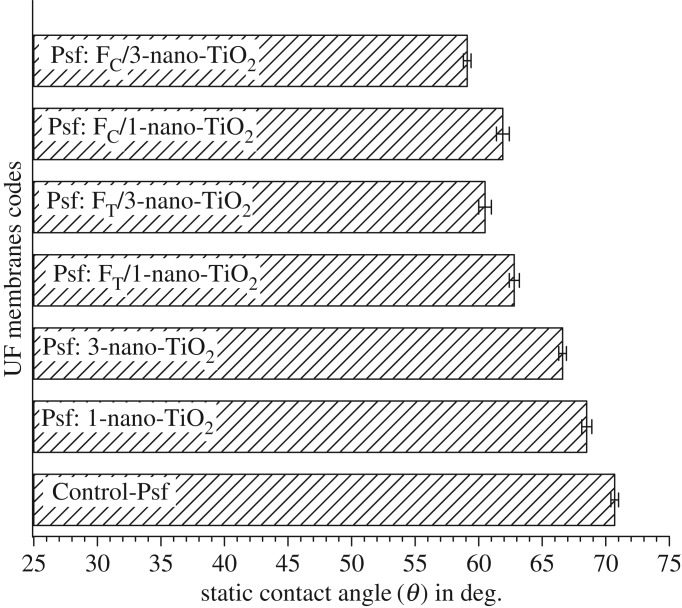
Hydrophilic characters of membranes: Control-Psf UF, Psf: nano-TiO_2_ UF, Psf: FT-nano-TiO_2_ UF and Psf: FC-nano-TiO_2_ UF.

The hydrophilic characters of the investigated membranes were assessed with respect to the probe solvent (water) to evaluate the role of organofunctionalized nano-TiO_2_ in modifying the surface chemistry of the MMMs. The variations in hydrophilic characters (figure 9) revealed that the impregnation of nano-TiO_2_ improves the hydrophilicity of the membranes and it is concomitant with the increasing concentrations of the organofunctionalized nano-TiO_2_. More particularly, the impregnations with 1 and 3 (w/w_Psf_)% loading of nano-TiO_2_ reduce the contact angle of Control-Psf UF (70.7°) by 2.2° and 4.1° in Psf: 1-nano-TiO_2_ UF and Psf: 3-nano-TiO_2_ UF, respectively. However, this decline in the contact angle appeared to be more significant by the impregnations of 1 and 3 (w/w_Psf_)% of F_T_-nano-TiO_2_ as the contact angle dropped more sharply by 7.9° and 10.2°, respectively. The MMMs derived from incorporating 1 and 3 (w/w_Psf_)% of F_C_-nano-TiO_2_ showed even further enhancement in the hydrophilicity with a decrease in the contact angle values by 8.8° and 11.6°, respectively, with respect to the Control-Psf UF. The impregnation of organofunctionalized nano-TiO_2_ influenced the direction of migration of the nanoadditives towards the skin surfaces of the membranes during the membrane synthesis, and it had a high distribution on the membrane surface owing to its affinity for the non-solvent ‘water’ [52]. The improvement in the hydrophilic nature corroborates the presence of hydrophilic $-{\text{SO}}_{3}^{-}{\text{H}}^{+}$ groups of the tethered organoligands, which could significantly enhance the efficacy of nano-TiO_2_ in modifying the intrinsic hydrophobic nature of the Control-Psf UF. The facilitated distribution of organofunctionalized nano-TiO_2_ within the Psf matrix implies that the modified nanoadditives not only influence the physico-chemical features of the membrane skin surface but also the skin layer porous pathways [53], which collectively contribute towards enhancing the hydrophilicity of the MMMs.

**Figure 10.**
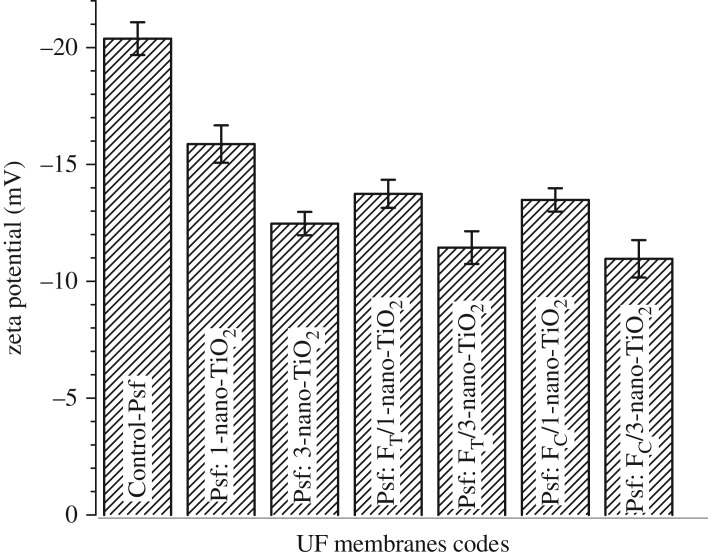
Electrokinetic features of membranes: Control-Psf UF, Psf: nano-TiO_2_ UF, Psf: FT-nano-TiO_2_ UF and Psf: FC-nano-TiO_2_ UF.

The electrokinetic features of the membranes, determined by tangential streaming potential measurements with respect to 10^−3^ M KCl solution, are represented in figure 10. The Control-Psf UF, with a matrix devoid of any dissociable functionality, showed a ζ potential of –20.38 mV, which is attributed to the effect of specific adsorptions of Cl^−^ ions from the electrolyte solution on the hydrophobic membrane surface [54]. However, the investigated MMMs offer different weak or strong sources of charges, which arise due to the non-covalently fixed –OH groups on the surface of nano-TiO_2_ or the $-{\text{SO}}_{3}^{-}{\text{H}}^{+}$ groups of the organoligands attached to the nano-TiO_2_. Therefore, the surfaces of the MMMs exhibit predominant contributions of the tethered functionalities in surpassing the tentative ionic adsorptions by the intrinsic character of the Control-Psf matrix. The charge-carrying sites of the mixed-matrix system bestow the surface conductivity to the membranes, and the extent and exposure of these sites through their varying distributions alter the electrokinetic features of the membranes’ surface [55]. This effect is reflected through the variations in the ζ potential of the MMMs, as the ζ potential changes from −15.87 to −12.47 mV for Psf: nano-TiO_2_ UF, −13.74 to −11.44 mV for Psf: F_T_-nano-TiO_2_ UF and −13.48 to −10.96 mV for Psf: F_C_-nano-TiO_2_ UF on impregnation of 1 and 3 (w/w_Psf_)% of nano-TiO_2_, F_T_-nano-TiO_2_ and F_C_-nano-TiO_2_, respectively. The decline in the ζ potentials of the MMMs compared to the Control-Psf UF indicates the altered electrokinetic events, which stem from the modified compositions of electrochemical double layers in the case of the mixed-matrix system. This influence is observed to be progressively pronounced on moving from Psf: nano-TiO_2_ UF to Psf: F_T_-nano-TiO_2_ UF and then to Psf: F_C_-nano-TiO_2_ UF. The enhanced impregnations of F_T_-nano-TiO_2_ and F_C_-nano-TiO_2_ seem to elevate markedly the skin layer conductivities of the respective membranes. This variation can emerge from the dragging of tangentially driven counter-ions from the hydrodynamic slipping plane (or plane of shear) to the bulk of the membrane's charged layer, through the hydrodynamically stagnant layer of counter-ions. The effective streaming currents of the membranes are thus supposed to be reduced due to such diffusive backflow of the streaming current [56], a phenomenon that is substantiated by the observed electrokinetic features, as manifested through the decline of the potential in the respective membranes.

**Figure 11.**
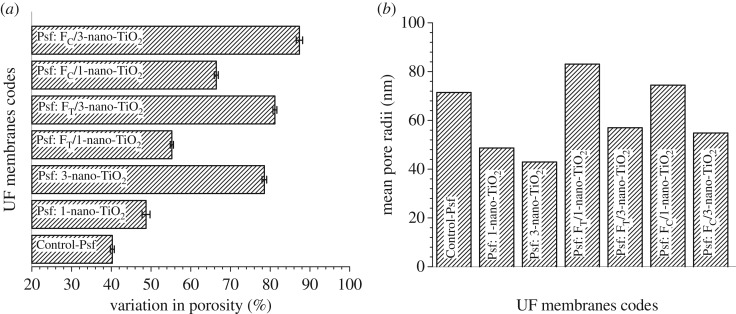
Variations in (*a*) porosities and (*b*) mean pore radii of membranes: Control-Psf UF, Psf: nano-TiO_2_ UF, Psf: FT-nano-TiO_2_ UF and Psf: FC-nano-TiO_2_ UF.

The variation in porosities of the MMMs as a function of the extent of nanoadditives is represented in figure 11*a*. A distinctive improvement in the porosities of the MMMs is evident from the estimated values, i.e. 40 ± 0.5% for the Control-Psf UF to 48 ± 1% for Psf: 1-nano-TiO_2_ UF, 55 ± 0.4% for Psf: F_T_/1-nano-TiO_2_ UF and then further to 66 ± 0.5% for Psf: F_C_/1-nano-TiO_2_ UF. This enhancement in the porosity becomes more pronounced when nanoadditives are impregnated at higher concentrations. The variations in porosities of the MMMs, i.e. 78 ± 0.6% for Psf: 3-nano-TiO_2_ UF**,** 81 ± 0.5% for Psf: F_T_/3-nano-TiO_2_ UF and 87 ± 0.8% for Psf: F_C_/3-nano-TiO_2_ UF further corroborate the aforesaid fact. The trends also imply that the sheaths of the organoligand–chromotropic acid remain more effective when compared with that of Tiron in tuning the electrostatic stabilization of the nano-TiO_2_ within the precursor dope solutions, and resultantly provide more porous mixed matrices in comparison to the former. An evaluation of the mean pore radii in the membranes (figure 11*b*) indicates that the membranes derived from higher loading of nano-TiO_2_ or organofunctionalized nano-TiO_2_ consist of significantly finer pores than those of the membranes with lesser loading. The estimated mean pore radii were found to vary as 49 to 43 nm for Psf: nano-TiO_2_ UF, 83 to 57 nm for Psf: F_T_-nano-TiO_2_ UF and 74 to 55 nm for Psf: F_C_-nano-TiO_2_ UF on impregnation of 1 and 3 (w/w_Psf_)% of nano-TiO_2_, F_T_-nano-TiO_2_ and F_C_-nano-TiO_2_, respectively. It is also noteworthy that the estimated porosities and the respective mean pore radii of the MMMs have an inverse relationship, i.e. the membranes with more porous features have smaller-sized pores and vice versa. The findings can be attributed to the incorporation of hydrophilic nanoadditives, which are expected to affect the mechanism of the phase separation process [53]. The NIPS process is known to be influenced by the thermodynamic, as well as kinetic variations that lead to either instantaneous or delayed demixing during the precipitation of casted polymer solutions [57,58]. The instantaneous demixing generally leads to a membrane with a highly porous substructure and a thin skin layer with fine pores, whereas the delayed demixing results in a membrane with a mildly porous substructure with a dense skin layer. It is thus hypothesized that the organofunctionalized nano-TiO_2_ could notably facilitate the rate of instantaneous demixing, i.e. the diffusive mass exchange between the solvent and the non-solvent, leading to membranes with high porosity and small-sized pores.

### 3.3. Study of mixed-matrix membranes ultrafiltration performances in terms of solute rejection and solvent flux behaviours

**Figure 12.**
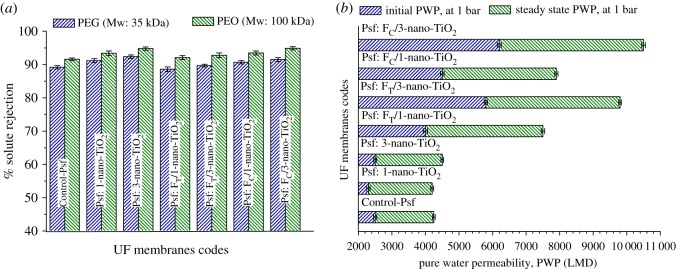
Macroscopical features, (*a*) % SR and (*b*) PWP of membranes: Control-Psf UF, Psf: nano-TiO_2_ UF, Psf: FT-nano-TiO_2_ UF and Psf: FC-nano-TiO_2_ UF.

The %SR and PWP, measured under a steady-state condition, reflecting the variations in macroscopical features of the MMMs are presented in figure 12*a* and *b*, respectively. The observations signify that the organofunctionalized nano-TiO_2_ induce noteworthy physico-chemical alterations in the MMMs. The %SR values for PEG and PEO were 89.2 and 91.6%, respectively, for Control-Psf UF. These values increased to 91.2, 93.4 and 92.4, 94.8% for Psf: 1-nano-TiO_2_ UF and Psf: 3-nano-TiO_2_ UF, respectively. However, the rejections of the solutes were slightly reduced to 88.6, 92.1 and 89.7, 92.8% for Psf: F_T_/1-nano-TiO_2_ UF and Psf: F_T_/3-nano-TiO_2_ UF, respectively. For Psf: F_C_/1-nano-TiO_2_ UF and Psf: F_C_/3-nano-TiO_2_ UF, the rejections of the solutes were 90.7, 93.5 and 91.5, 94.9%, respectively. The analysis of steady state PWP of the membranes indicates a gradual change from 1750 LMD for Control-Psf UF to 1920 LMD for Psf: 1-nano-TiO_2_ UF and then to 2000 LMD for Psf: 3-nano-TiO_2_ UF. The impregnations of organofunctionalized nano-TiO_2_, *viz*. F_T_-nano-TiO_2_ and F_C_-nano-TiO_2_, within the Psf matrices resulted in an obvious improvement in PWP. The flux enhancements are manifested through the PWP values: 3500, 4000 and 3400, 4300 LMD for Psf: F_T_/1-nano-TiO_2_ UF, Psf: F_T_/3-nano-TiO_2_ UF and Psf: F_C_/1-nano-TiO_2_ UF, Psf: F_C_/3-nano-TiO_2_ UF, respectively.

The trends obtained for the flux and rejection behaviours are in good accordance with the discussed variations in the porosities and mean pore radii of the investigated membranes. As the impregnation of nano-TiO_2_ as well as the organofunctionalized nano-TiO_2_ influences the mechanism of pore formation during the NIPS process by altering the in-diffusion rate of the non-solvent and the out-diffusion rate of the solvent, the resultant fine pores on the skin layer of the membrane, therefore, enhance %SR for the MMMs. The increase in PWP of the Psf: 1-nano-TiO_2_ UF corroborates the fact of enhanced surface roughness as well as hydrophilicity. However, the extent of increase in PWP on impregnation of pristine nano-TiO_2_ at a higher weight fraction seems not to be significant, which can be attributed to the observed aggregation at a higher concentration of nano-TiO_2_, leading to the presence of clusters of nano-TiO_2_ in the porous membrane matrix. It can be anticipated that the modified nanoadditives play a potential role in controlling the formation of finer pores in the skin region of the membranes while restoring better uniformity of distribution within the polymer matrices. The aggregation tendency of the nanoadditives at a higher concentration and the consequent effect on membrane performance are restricted to a large extent by the deployment of the functionalized nanoparticles sheathed by organoligands. However, mass transport in the UF membrane is mainly facilitated by hydrodynamic interactions; there could be some simultaneous and synergistic contributions from electrostatic interactions in the case of mixed-matrix systems. The implanted charged sites (e.g. $-{\text{SO}}_{3}^{-}{\text{H}}^{+}$ in this case) present over the nano-TiO_2_ surface could support reasonable electrostatic interactions with the diffusing solvent molecules, and thereby may lead to an improvement in the hydrodynamic flow through the porous pathways of the membrane [59]. The MMMs having high porosities and amendable pore sizes may offer facilitated transport features such as better retention ability with high productivity.

### 3.4. Investigation of mixed-matrix membranes antifouling properties

**Figure 13.**
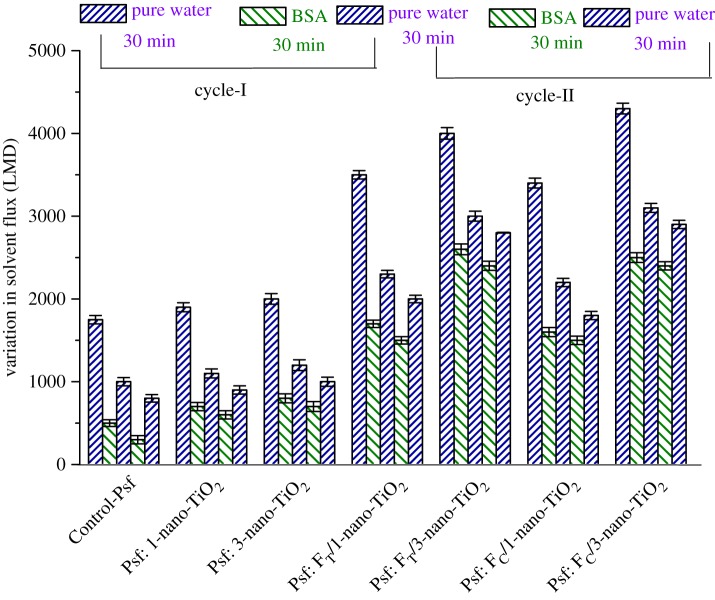
Time-dependent variation in solvent fluxes of membranes during the ultrafiltration of BSA solution following two-step cyclic operation, which involves three times of pure water ultrafiltration and two times of BSA solution ultrafiltration. After each ultrafiltration of BSA solution, cleaning through pure water flushing was conducted.

To understand and comprehend the effect of incorporation of organofunctionalized nano-TiO_2_ in the modulation of the antifouling property of the MMMs, a two-step cyclic UF test was performed using BSA as a model protein. The membrane fouling is influenced primarily by the hydrodynamic factors such as the permeation drag of the foulants, i.e. the movement of BSA molecules from the bulk of the solution to the membrane surface and the diffusive back transport of BSA in the reverse direction. The secondary interfacial non-covalent (hydrophobic) interaction between the polymeric surface and the penetrating foulants also influences the fouling tendency of the membrane. Thus, the fouling behaviours of the membranes were evaluated under similar hydrodynamic conditions to get an insight into the effect of the impregnation by nanoadditives. The time-dependent flux variations and the determined FRRs are presented in figure 13 and table 5, respectively.

**Table 5.** Variations in antifouling properties of investigated membranes in terms of flux recovery ratios (FRRs) and resistances towards solvent fluxes caused by total fouling (*R*_t_), reversible fouling (*R*_r_) and irreversible fouling (*R*_ir_).

	Cycle-I				Cycle-II			
UF membranes codes	FRR (%)	*R*_r_ (%)	*R*_ir_ (%)	*R*_t_ (%)	FRR (%)	*R*_r_ (%)	*R*_ir_ (%)	*R*_t_ (%)
Control-Psf	57.1	28.6	42.9	71.5	45.7	22.9	54.3	77.2
Psf: 1-nano-TiO_2_	57.9	21.1	42.1	63.2	47.4	15.8	52.6	68.4
Psf: 3-nano-TiO_2_	60.0	20.0	40.0	60.0	50.0	15.0	50.0	65.0
Psf: F_T_/1-nano-TiO_2_	65.7	17.1	34.3	51.4	57.1	11.4	42.9	54.3
Psf: F_T_/3-nano-TiO_2_	75.0	10.0	25.0	35.0	70.0	7.5	30.0	37.5
Psf: F_C_/1-nano-TiO_2_	64.7	17.7	35.3	53.0	55.9	11.8	44.1	55.9
Psf: F_C_/3-nano-TiO_2_	72.1	13.9	27.9	41.8	65.1	9.3	34.9	44.2

It is obvious from figure 13 that, for all the membranes, the stabilized solvent fluxes decline with the filtration of the protein solution compared to that of pure water permeation. Owing to their relatively low diffusion coefficient, the diffusive back transport of the BSA molecules is overcompensated by their convective transport towards the membrane surface, which results in the enhancement of BSA concentration at the membrane surface leading to a concentration polarization. The process of cross-flow UF is a collective contribution of shear tangential flow and steady transverse flow, wherein the former remains predominant in overcoming the effect of concentration polarization, while the latter profoundly contributes to the concentration polarization. Furthermore, the BSA molecules prefer to get accumulated on the membrane surface to minimize the Gibbs free energy during their transverse flow, and progressively reach a thermodynamic equilibrium by establishing a concentration polarization [60]. The resistive layers, consequently formed in all the membrane surfaces, thereby induce hydrodynamic resistance to the solvent permeation. The flux decline further promotes the notorious effect of membrane fouling as the penetrating BSA molecules gradually coalesce at the surface pores causing pore blockage as well as the formation of cake layers, and that prevents solvent permeation to a large extent [1,6].

During the first cycle (Cycle-I), the FRR of the membranes (table 5) increased from 57.1% for Control-Psf UF to 57.9 and 60% for Psf: 1-nano-TiO_2_ UF and Psf: 3-nano-TiO_2_ UF; 65.7 and 75.0% for Psf: F_T_/1-nano-TiO_2_ UF and Psf: F_T_/3-nano-TiO_2_ UF; and 64.7 and 72.1% for Psf: F_C_/1-nano-TiO_2_ UF and Psf: F_C_/3-nano-TiO_2_ UF, respectively. This trend reveals that the membranes comprising the nanoadditive-nano-TiO_2_ exhibit slightly better antifouling characteristics than the Control-Psf UF. This is conferred by the improved hydrophilicity of the resultant MMMs, wherein the hydration layers impede the accumulation of the hydrophobic BSA molecules to some extent [17,61]. In addition, the impact of the nanoadditives became more pronounced when the F_T_-nano-TiO_2_ and F_C_-nano-TiO_2_ were employed at different concentrations in amending the membrane surfaces. The peripheral presence of the $-{\text{SO}}_{3}^{-}{\text{H}}^{+}$ groups, tethered via the organoligands at the surface of nano-TiO_2_, and the resultant uniformity in the distributions of the modified nanoadditives within the Psf matrices impart strong long-range repulsive forces. This also leads to the formation of more stable and fouling-resistant hydration layers, which inhibit the onset of fouling (concentration polarization) by preventing the possible formation of a concentrated stationary layer of the foulant at the upstream side of the membranes. The variations noticed in the FRR of the second cycle (Cycle-II), i.e. 45.7% for Control-Psf UF to 47.4 and 50% for Psf: 1-nano-TiO_2_ UF and Psf: 3-nano-TiO_2_ UF; 57.1 and 70.0% for Psf: F_T_/1-nano-TiO_2_ UF and Psf: F_T_/3-nano-TiO_2_ UF; and 55.9 and 65.1% for Psf: F_C_/1-nano-TiO_2_ UF and Psf: F_C_/3-nano-TiO_2_ UF, respectively, substantiate the role of organoligands in altering the protein–membrane interaction and leading to better antifouling properties.

The overall fouling process is a cumulative effect of reversible and irreversible fouling events. Therefore, the antifouling properties of the membranes were further investigated by analysing the resistances towards solvent fluxes caused by total fouling (*R*_t_), reversible fouling (*R*_r_) and irreversible fouling (*R*_ir_) [39,62]. The effect of irreversible fouling seems to be more pronounced in reducing the solvent flux when compared with the reversible fouling because the latter can be well controlled by simple hydraulic cleaning, whereas the former is an outcome of a strong adhesion of the foulants to the membrane surface. It can be seen in table 5 that, during both cyclic UF operations, the *R*_t_ value declines sharply with the use of F_T_-nano-TiO_2_ and F_C_-nano-TiO_2_; the component values of *R*_t_, i.e. *R*_r_ and *R*_ir_, reduce successively for Psf: F_T_/1-nano-TiO_2_ UF, Psf: F_T_/3-nano-TiO_2_ UF and Psf: F_C_/1-nano-TiO_2_ UF, Psf: F_C_/3-nano-TiO_2_ UF to a significant extent. The improvement in antifouling characteristics of the MMMs using functionalized nano-TiO_2_ is more pronounced than that of the pristine nano-TiO_2_ systems. A comparison of *R*_r_ and *R*_ir_ implies that the organofunctionalized nanoadditives endow a better antifouling behaviour at higher concentrations, whereas even a higher loading of the pristine nanoadditives does not exert much effect. The results of FRRs and the fouling-resistant behaviours confirm the capability of the organofunctionalized nanoadditives, *viz.* F_T_-nano-TiO_2_ and F_C_-nano-TiO_2_, to be potential contenders in the development of antifouling MMMs.

## 4. Conclusion

The precursor anatase nano-TiO_2_ was modified by facile chemisorption processes using two different potential organoligands, Tiron and chromotropic acid, to synthesize organofunctionalized nanoadditives, F_T_-nano-TiO_2_ and F_C_-nano-TiO_2_, respectively. The functionalization processes led to surface-tailored nano-TiO_2_ with charged sites ($-{\text{SO}}_{3}^{-}{\text{H}}^{+}$). The MMMs, *viz.* Psf: F_T_-nano-TiO_2_ UF and Psf: F_C_-nano-TiO_2_ UF comprising Psf and the organofunctionalized nanoadditives, were prepared using various compositions following the NIPS technique. The physico-chemical characteristics of the membranes were modulated by nanoadditives, which resulted in mixed-matrix systems with enhanced surface hydrophilicity, increased porosity and altered electrokinetic features. The morphology and topography of the MMMs were improved by incorporating the organofunctionalized nanoadditives within the Psf matrix. The flux and solute rejection behaviours indicated an enhanced performance of the MMMs, as they showed very high solvent throughputs, i.e. 3500–4000 and 3400–4300 LMD for F_T_-nano-TiO_2_ and F_C_-nano-TiO_2_, respectively, at 1 bar of TMP, without any notable deterioration in their solute rejection capability.

The analysis of antifouling activity, determined by a two-step cyclic filtration process with BSA solution, demonstrated that both the classes of the MMMs, amended by impregnating F_T_-nano-TiO_2_ and F_C_-nano-TiO_2_, exhibited superior FRRs when compared with the Control-Psf as well as the pristine nano-TiO_2_-based membranes. Furthermore, the deteriorating effect of reversible as well as irreversible fouling was considerably reduced by the use of both organofunctionalized nanoadditives, indicating a better prospect for the reusability of the MMMs for robust UF applications. Such a membrane system with superior fouling resistance characteristics coupled with enhanced solvent throughput can potentially lead to an energy-efficient and environment-friendly UF process having diverse industrial perspectives, especially for applications related to waste water treatment.

## Data accessibility

The details of the research materials pertaining to this work have been provided in the manuscript itself.
